# Use of a decision-analytic model in a health technology assessment: beyond measuring value for money

**DOI:** 10.1186/2045-4015-2-15

**Published:** 2013-04-22

**Authors:** Sun-Young Kim

**Affiliations:** 1 Division of Management, Policy and Community Health, University of Texas School of Public Health, San Antonio Regional Campus, 7411 John Smith Drive, Suite 1100, San Antonio, TX 78229, USA

## Abstract

The well-designed, model-based cost-utility analysis by Ginsberg and colleagues provides useful information on the value for money of universal GBS screening in Israel. An extended application of the model-based approach used in the study could provide policymakers additional practical information on the budget impact of a potential universal GBS screening program. Such an approach could also be used to guide future research priorities in the prevention of GBS in Israel, by measuring the value of seeking further information to reduce the uncertainty in the cost-effectiveness of universal GBS screening.

This is a commentary on http://www.ijhpr.org/content/2/1/6.

## 

Policymakers are confronted with a variety of policy questions in making resource allocation decisions among health interventions that target diverse groups of patients or subpopulations. These questions range from efficiency to equity, and often involve the consideration of a society’s core values. Due to the ever-increasing economic burden of health care globally and the availability of new (but highly expensive) technologies, one of the most frequently asked questions by policymakers in regards to health interventions is whether they provide good value for money relative to alternative interventions. In the previous issue, the well-designed, model-based cost-utility analysis conducted by Ginsberg and his colleagues [1] provides an example of studies that can answer such a question.

In the study, Ginsberg and colleagues attempt to address the question of whether Israel should expand its coverage of preventive screening against group B streptococcal (GBS) infection to all pregnant women [1]. In doing so, they take the framework of cost-utility analysis (or cost-effectiveness analysis, which is often interchangeably used, but encompasses both types of analyses with and without utility assessment of health outcomes). They then assess the potential health and economic impact of universal screening (taking a vaginal-rector culture for GBS screening from all pregnant women at 35–37 weeks of gestation) compared to the current practice in Israel of risk-based screening (performing a culture screening in mothers with known risk factors only) in terms of additional costs per quality-adjusted life year gained.

In conducting a health technology assessment, it is crucial to choose the right approach and methods according to the nature of the questions being asked and policy goals. It would be ideal if well-designed, rigorously conducted clinical trials could provide data on both costs and effectiveness of an intervention under consideration. Unfortunately, for multiple reasons (e.g., prohibitively high costs or ethical concerns), such rigorous primary research is not feasible for many health technology assessment studies [2]. When there are not enough high quality datasets with which to assess the costs and effectiveness profiles of an intervention, a decision-analytic simulation model can serve as a useful tool to synthesize evidence and imperfect information [3]. More and more recent economic evaluation studies or health technology assessments take a model-based approach, supplementing the results obtained from empirical studies. There are various types of models, ranging from a simple, less costly, transparent model to a complex, costly, but more flexible one [4]. When conducting a model-based study, it is also crucial to choose the right type of decision-analytic model, taking into account the nature of the disease being simulated and the trade-offs among different types of models [5].

In conducting an *ex ante* evaluation of universal GBS screening, Ginsberg and colleagues take a model-based approach [1]. They develop a spreadsheet model (implicitly based on a decision-tree structure) and synthesize the best available data into the model to project the costs and health outcomes of the two alternative screening strategies for preventing the burden of GBS disease in Israel. Their findings suggest that universal GBS screening would offset a majority of the program costs for expanding its coverage by effectively reducing the burden of GBS disease and thus saving medical costs for treating GBS disease in infants.

A model-based study inevitably requires assumptions about the costs, effectiveness of interventions, and disease burden [3,6]. Accordingly, the quality of a health technology assessment using a model is only as good as the quality of the assumptions and data synthesized into the model that provides the information.

Despite these inherent limitations, a model-based approach has several strengths [3,6]. In this light, the model-based approach used in Ginsberg et al.’s study has the potential to provide policymakers additional practical information beyond the cost-effectiveness profile of universal GBS screening that they might find useful in making decisions about whether to adopt a universal screening program.

First of all, a decision-analytic model can be set up (by being assigned probability distributions to model parameters) so that it can conduct a probabilistic analysis based on Monte Carlo simulations. Such a probabilistic decision-analytic model can then be used to perform a more comprehensive type of sensitivity analysis (e.g., a probabilistic sensitivity analysis that varies all key parameters at the same time). In Ginsberg et al.’s study, the deterministic one- and two-way sensitivity analysis results note that, while the base-case results are very cost-effective, the results are very sensitive to some of the key parameters (such as GBS prevalence, the proportion of meningitis cases leading to long-term disabilities, and the probability of intrapartum antibiotic prophylaxis-related anaphylaxis). This may warrant a more comprehensive sensitivity analysis that varies all key variables at the same time. Ginsberg et al.’s study could use their model to conduct a probabilistic sensitivity analysis by assigning distributions to key parameters and conducting a second-order Monte Carlo simulation in which all key parameter values are sampled from the distributions. The simulated outcomes then can be summarized in some form of visual presentation, in order to provide more accessible and comprehensive information on the uncertainties about the outcomes of a universal screening. For example, a cost-effectiveness acceptability curve can show the probability that universal GBS screening would be cost-effective relative to the current risk-based screening at varying levels of threshold cost-effectiveness ratio [7].

Further, once a decision-analytic model is set up for a probabilistic analysis, the same probabilistic model can be used to conduct another type of uncertainty analysis, a value of information (VOI) analysis. VOI analysis is an analytic framework that can calculate the value of seeking further information to support a decision [8]. For example, given the uncertainties surrounding some key parameters in Ginsberg et al.’s study, policymakers may want to know whether further research is needed to reduce uncertainty about the parameters and whether better information on the parameters would lead to a different conclusion about the optimal strategy for preventing GBS disease in Israel. In this case, a probabilistic decision simulation model can be used to perform a VOI analysis and can provide the value of reducing the uncertainty associated with the choice of universal GBS screening through additional research.

Another useful application of a model-based approach is that the same decision-analytic model can be used to project the budget impact of the potential adoption of a health intervention under consideration, which might provide additional practical information for policymakers [9]. The budget impact analysis is performed from the perspective of a payer and provides the financial requirements (by considering financial costs only, not economic costs) for adopting a new intervention over a typical period of 1–5 years, so that payers can compare the amount of financing required with the size of the available budget. Given Israel’s unique system of choosing health interventions under an annual budget constraint, policymakers might find this type of additional information particularly useful.

Does the study by Ginsberg and colleagues answer their stated question “Should Israel screen all mothers-to-be to prevent early-onset of neonatal group B streptococcal disease?” Assuming that the question implicitly focuses on a value for money (that is, based on cost-effectiveness grounds only), the answer may be yes. However, it is important to note that cost-utility analysis or cost-effectiveness analysis on their own cannot answer all priority-setting questions [10], and thus cannot conclusively answer the question of whether the decision makers would be best to adopt a universal screening strategy based on the findings of the study. Future health technology assessments should be conducted, keeping in mind this inherent restriction on the types of questions the cost-utility analysis framework can answer. Thus, by developing sophisticated decision-analytic methods (including the extended use of a decision-analytic model), researchers are positioned to best inform policymakers on the important types of questions to which their methods are suited.

## Competing interests

The author declares that she has no competing interests.

## Authors’ information

Sun-Young Kim, PhD, is an Assistant Professor at the Division of Management, Policy and Community Health at the University of Texas School of Public Health. She is also affiliated with the Center for Research to Advance Community Health (ReACH), San Antonio, TX, USA.
